# The complete mitogenome of the live-bearing fish *Xenotoca variata* (Bean, 1887) (Actinopterygii: Goodeidae)

**DOI:** 10.1080/23802359.2017.1289350

**Published:** 2017-02-23

**Authors:** Diushi Keri Corona-Santiago, Carolina Galván-Tirado, Francisco Javier García-De León, Ignacio Doadrio, Omar Domínguez-Domínguez

**Affiliations:** a Departamento de Biodiversidad y Biología Evolutiva, Museo Nacional de Ciencias Naturales, Madrid, Spain;; b Laboratorio de Genética para la Conservación, Centro de Investigaciones Biológicas del Noroeste, S.C, La Paz, Mexico;; c Laboratorio de Biología Acuática, Facultad de Biología, Universidad Michoacana de San Nicolás de Hidalgo, Morelia, Mexico

**Keywords:** Live-bearing, Goodeinae, Central Mexico, mitogenome

## Abstract

The live-bearing fish *Xenotoca variata* is representative of the viviparous Goodeinae subfamily (Goodeidae) from central Mexico. The mitogenome of the *X. variata* consist of 37 genes in 16,462 bp. Comparing with *X. eiseni*, the most related of the mitogenomes included, an identity of 91.1% was found and *trna-met* duplication in *X. eiseni* is absent in *X. variata*. The mitogenome provide important information for future studies in evolution of the live-bearing subfamily.

The species *Xenotoca variata* (Bean 1887), is representative of the live-bearing subfamily Goodeinae (Goodeidae) and is distributed in central Mexico: in the Middle Lerma River, Zacapu basin, Cuitzeo Lake, Chapala Lake, Pánuco and Aguanaval basins (Domínguez-Domínguez 2008). Goodeines have been a model for the study in evolution, due their peculiar characteristics of breeding strategies and embryo development. Important genetic divergences (Domínguez-Domínguez et al. 2010; Corona-Santiago & Domínguez-Domínguez 2013), sexual selection (Moyaho et al. 2004; Ritchie et al. 2007), substantial phenotypic plasticity (Fitzsimons 1972; Macías-Garcia 1998) have been observed among *X. variata* populations. Hence, the aim of this work is the characterization of mitochondrial genome of *Xenotoca variata*, which could provide relevant information for future studies.

For mitogenome sequencing we use a sample tissue (pectoral fin) of *Xenotoca variata* from Huingo spring (19°54′44.0″N 100°50′00.3″W), Cuitzeo basin, and were storage in the Colección de Peces de la Universidad Michoacana-UMSNH, Mexico (Voucher specimen: CPUM-7031). Briefly DNA was sheared using a Covaris S2 (Woburn, MA) ultrasonicator, and Illumina (Illumina, San Diego, CA) adapters were ligated on using methods derived from Fisher et al. (2011), but using adapters equivalent to Illumina TruSeq with 10nt indexes (Faircloth & Glenn 2012). Genomic DNA was subjected to sequencing at the Georgia Genomics Facility (University of Georgia). Reads quality was analyze using FastQC (Andrews 2010), adapters and poorly quality sequences were trimmed using Trimmomatic v0.36 (Bolger et al. 2014) to assembly using SOAPdenovo2 (Luo et al. 2012). Genome annotation was performed using MitoAnnotator (Iwasaki et al. 2013) but the position of all tRNA genes was confirmed using tRNAscanSE v1.21 (Schattner et al. 2005). Phylogenetic reconstruction was performed under Neighbour-Joining analysis including 14 species of the Cyprinodontiformes order available on GenBank. The analysis was conducted with a full alignment built in MAFFT v7.222 (Katoh et al. 2002).

The circular mitogenome of *Xenotoca variata* (GenBank accession: KY471393) consists of 37 genes in 16,511bp (13 protein-coding genes, 2 rRNA genes and 22 tRNA genes) with 12 intergenic spacer sequences (of 1–37bp). The base composition of the genome was as follow: A = 28.7%, C = 26.8%, G = 15.3% and T = 29.2% (GC-rich = 42.1%). Comparing with *X. eiseni*, the most related of the mitogenomes included in Genbank (Figure 1), an identity of 91.1% was founded and the duplication of *trna-met* gene in *X. eiseni* is absent in *X. variata*. The number of nucleotide differences between both mitogenomes is 1 206bp corresponding to 7.3% of divergence. The complete mitochondrial genome of *X. variata* provides relevant information to posterior genetic and evolutionary studies of *Xenotoca* genus and the Goodeidae family.

**Figure 1.**
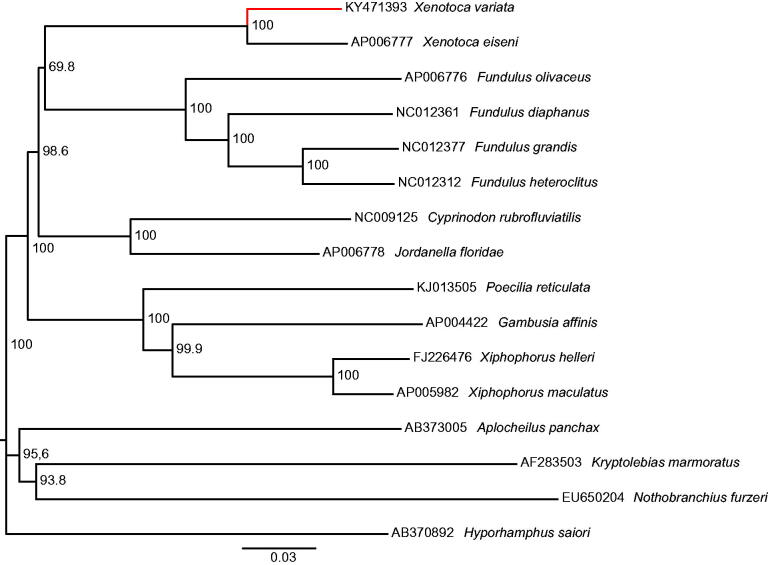
Sample validation of *Xenotoca variata* based on Neighbour-Joining tree (1000 bootstrap replicated using uncorrected *p*-distances) with 15 species of the Cyprinodontiformes order.
